# Prevalence of illicit alcohol consumption and associated factors among adolescents in selected communities of Lusaka, Zambia

**DOI:** 10.1371/journal.pone.0336208

**Published:** 2026-01-16

**Authors:** Dhally M. Menda, Tom Achoki, Bristol M. Ntebeka, Rosemary K. Zimba, Catherine M. Mulikita, Angela Rizzo, Maynards C. Tembe, Sharon Nkwemu, Rodgers Chilyabanyama, Karen Sichinga, Michael Kachumi, Joel Msafiri Francis, Choolwe Jacobs

**Affiliations:** 1 Department of Health Programs, Churches Health Association of Zambia, Lusaka, Zambia; 2 Chreso University and Lusaka University, Lusaka, Zambia; 3 ABInBev Foundation, New York, United States of America; 4 Africa Institute for Health Policy, Nairobi, Kenya; 5 Department of Epidemiology and Biostatistics, School of Public Health, University of Zambia, Lusaka, Zambia; 6 Churches Health Association of Zambia, Lusaka, Zambia; 7 University of the Witwatersrand, Johannesburg, South Africa; 8 Women in Global Health, Lusaka Zambia; University of Pretoria, SOUTH AFRICA

## Abstract

**Introduction:**

Illicit alcohol (illegally brewed alcohol) consumption among adolescents is an increasing public health problem worldwide, with major impacts on individuals, families, and communities. It’s effects may lead to blindness, poisoning, infection, and other health problems. In Zambia, evidence on the burden of illicit alcohol intake and associated factors is limited, particularly in socioeconomically deprived communities. This study estimated the prevalence and examined the socio- demographic, socio-cultural and socio-economic factors that influence illicit alcohol consumption among adolescents between 10–17 years old in selected unplanned settlements in the city of Lusaka, Zambia.

**Methods:**

A cross-sectional study was conducted in December 2023, among 331 adolescents who reported alcohol use in 24 selected densely populated peri-urban communities of Lusaka, Zambia. Total enumeration was employed to select participants who met the age criteria and were enrolled in the Screening, Brief Intervention, and Referral to Treatment (SBIRT) program. Face-to-face questionnaire interviews were conducted to collect data on socio-demographic, socio-cultural, socio-economic, and illicit alcohol intake characteristics. Descriptive statistics and graphical presentations were generated, and an investigator-led stepwise multiple logistic regression analysis was performed to identify factors influencing illicit alcohol consumption, with variables with p-values >0.2 removed from the model. Data were analyzed using STATA version 16 MP, and statistical significance was set at p < 0.05.

**Results:**

The prevalence of illicit alcohol consumption among adolescent alcohol users in the selected Lusaka communities was 42.9% (142/331), with the majority being male (75%). The median age of participants was 16 years (IQR: 15–17). Most adolescents (98.8%, 327/331) had attended school, though only 32% (102/331) had reached secondary level. A large proportion (77.3%, 256/331) lived with their biological parents, while 73.7% (244/331) belonged to large households (more than 10 members). After adjusting for potential confounders, males were more than twice as likely to engage in illicit alcohol intake compared to females (aOR = 2.08, 95% CI: 1.22–3.55, p = 0.005). Conversely, unemployed adolescents were significantly less likely to consume illicit alcohol compared to their employed peers (aOR = 0.52, 95% CI: 0.28–0.90, p = 0.048).

**Conclusion:**

Illicit alcohol intake among adolescents in peri-urban communities of Lusaka, Zambia, is high and linked to a broad range of risk factors, particularly sex and employment status. Early initiation of comprehensive, multisectoral, community-based strategies with active stakeholder involvement may help mitigate this growing public health problem.

## Introduction

Consumption of Illicit alcohol among adolescents is an increasing public health problem worldwide, leading to several health problems including blindness, poisoning and infection [1,2]. This problem is amplified by the prevalence of alcohol consumption among adolescents of non-legal drinking age [3,4,Trucco *et al.*, 2014,^5^], with approximately 43% of the world’s adolescent population above 15 years have consumed alcohol at some point [WHO 2018]. Underage alcohol consumption is a major public health concern throughout Africa [2,6] including Zambia [7]. Research shows that adolescents’ illicit alcohol consumption has a major impact on individuals, families, and communities, as its effects are cumulative, contributing to costly social, physical, and mental health problems [8,9]. Illicit alcohol consumption among adolescents can also lead to both health and socioeconomic challenges [10,11].

In this study, illicit alcohol consumption is defined as the use of alcoholic beverages produced illegally, outside of approved and regulated processes, by adolescents. Owing to the absence of quality control in their production, these beverages often contain harmful substances such as methanol, fire extinguisher powder, or rat poison, which can lead to blindness, organ damage, or even death [1,2]. Additionally, as illegal alcohol can be less expensive and easier for minors to access than authorized beverages, it is often associated with underage drinking [6]. This is particularly concerning as young people are more vulnerable to the harmful effects of alcohol, with an increased risk of accidents, injuries, and delayed brain development [12,13].

Findings from studies across several African countries reveal a high prevalence of alcohol consumption among adolescents, with strong links to risky behaviors such as unprotected sex and violence [14,15,5,16]. Factors associated with illicit alcohol use among underage is not well documented. However, some studies have identified demographic factors such as gender, age, living arrangements, and peer pressure as key contributors [13,6]. Peer pressure in particular, has been shown to strongly influence alcohol intake among adolescents who drink to fit in with their social group [17,7]. Other studies argue that many adolescents may engage in risky behaviors due to ignorance about the negative consequences of alcohol consumption [18]. Easy access to alcohol through older friends or family members has also been reported as associated with alcohol use among adolescents [19,14,15,20,5]. In Africa, illicit alcohol consumption patterns among adolescents is further shaped by cultural norms, socioeconomic disparities and limited access to education and healthcare [21]. Addressing these hazards associated with underage and illicit alcohol consumption and promoting safer settings is crucial [13,6].

In Zambia, few studies have examined the prevalence of alcohol use and its associated factors, with most focusing on primarily on adult populations [15,22]. Evidence on the prevalence of illicit alcohol use and its determinants among underage adolescents is scarce. Yet, understanding the burden and associated factors is crucial for designing responsive strategies to mitigate this growing public health concern. This study therefore aimed to assess the prevalence of illicit alcohol use and its associated factors among adolescents aged 10–17 years in selected communities of Lusaka, Zambia.

## Methods

### Study design

This research employed a cross-sectional study design, conducted in December 2023 among beneficiaries of the Churches Health Association of Zambia (CHAZ) Screening Brief Intervention Referral to Treatment (SBIRT) program in Lusaka.

### Study setting

The study was conducted in selected densely populated peri-urban communities of Lusaka, Zambia, selected to represent diverse socio-economic strata. The vibrant capital city of Zambia, Lusaka, reflects the nation’s rich cultural diversity. Its dense population is shaped up of a dynamic mix of cultures and customs [8]. Lusaka, however, also has to deal with the negative effects of growing urbanization. There are many young people living in the city, including homeless youths (often known as street kids). Lusaka offers a wide range of activities, from vibrant markets and historical buildings, to makeshift bars and alcohol sale points, despite the public health concerns these pose. Given its high population density, and allegations of widespread alcohol consumption, the city is an appropriate place to investigate the factors affecting this issue of public health concern [18].

Specifically, the study was conducted in 24 communities, supported by the CHAZ SBIRT program [CHAZ, 2023]. These communities included Bauleni, Chainda, Chazanga, Chibolya, Chilenje Compound, Chunga, Garden, George Compound, George stage 2, Kabwata, Kalikiliki, Kalingalinga, Kamanga, Kamwala, Kasisi, Kaunda square, Mahopo, Malata, Mandevu, Matero, Misisi, Mtendere compound, Ngombe, and Shardrecks. These sites were chosen because they are unplanned settlements in the city of Lusaka. Most heads of households in these communities are not in formal employment and they depend on small businesses, i.e., selling on the streets and in the market for their income [23,24, 25].

### Sampling of study participants

We included all the adolescents between the ages of 10–17 years adolescents, residing within the communities being serviced by the SBIRT program, and who were consuming alcohol. The CHAZ SBIRT program provides screening brief interventions in public health facilities and their surrounding communities,and referral to treatment for alcohol abusers needing more care. This study employed a targeted approach, focusing on adolescent alcohol consumers, within the program. All adolescents identified as consuming alcohol, and further listed within the SBIRT (Screening, Brief Intervention and Referral to Treatment) program’s database from these communities were included. This total enumeration approach ensured that we captured every eligible participant from the targeted population, minimizing sampling bias and allowing for a comprehensive representation of underage alcohol consumers within the selected communities. All individuals who reported consuming alcohol and were between the ages 10–17 years, were eligible.

### Study variables and measurements

Data were collected using a semi-structured interviewer-administered questionnaire adapted from the Zambia National Demographic Health Survey questionnaire, and other validated behavioral survey tool (Higgins- Biddle and Babor, 2018,Kidd *et al.*, 2022,Boschuetz and German, 2023,ZDHS, 2024]. Variables included in the analysis were assessed as follows:

#### Outcome variables.

- Illicit alcohol use: Defined as consumption of unregulated or informally produced alcoholic beverages (e.g., Kachasu, Tujilijili, and other illegally-brewed spirits) within the past 12 months.

#### Explanatory Variables.

- Age: Self-reported in years and recorded as a continuous variable; for descriptive and regression analysis, categorized into early adolescence (10–12 years), middle adolescence (13–15 years), and late adolescence (16–17 years), based on WHO adolescent age-group classification.
- Sex: Biological sexual orientation. Self-reported as male or female.
- School attendance: Recorded as ever attended school (Yes/No) and currently in school (Yes/No).
- Level of education: Categorized into Grade 1–7, Grade 8–9, and Grade 10–12.
- Employment status: Self-reported as employed (any paid work in the last month) or unemployed.
- Household composition: Living with both biological parents (Yes/No); head of household identified as father, mother, grandparent, or guardian.
- Head of household employment: Categorized as formal employment, informal employment, or unemployed.
- Family size: Number of household members, categorized as small (<5), medium (5–10), or large (>10).
- History of imprisonment: Respondents asked if they had ever been imprisoned or detained (Yes/No).
- Engagement in physical fights: In the past 12 months, have you been involved in a physical fight? (Yes/No).
- Drug use: Self-reported use of illicit drugs (e.g., marijuana, glue, inhalants) in the past 12 months (Yes/No).
- Sexual activity: Ever had sexual intercourse (Yes/No).
- Drinking frequency: Frequency of alcohol consumption in the past 12 months, categorized as daily, weekly, monthly, or yearly.

Variable selection for regression analysis was based on both empirical significance (based on investigator-led step-wise regression) and theoretical relevance as supported by prior literature and the Social Ecological Model [Kilanowski, 2017].

### Data collection

A structured, pre-tested questionnaire was administered, through face-to-face interviews to collect primary data. Data collection was conducted electronically using tablets with the KoboCollect software. The questionnaire covered questions on demographic characteristics, alcohol consumption patterns, socio-economic factors, and health-related behaviors specific to underage alcohol users. Interviews were conducted by young (20–30 years old), trained and experienced research assistants. We paid particular attention to making sure that each participant was paired with a research assistant they would be comfortable speaking to, based on age and sex. The interviews were conducted in private areas such as community hall to ensure participant confidentiality. Data security was maintained, by storing it on a password-protected computer, with restricted access.

### Data management

Data was captured within Kobo Collect on android tablets, and exported to excel. The dataset underwent a thorough cleaning to ensure completeness and consistency. This involved checking for missing values, inconsistencies, and logical errors. Subsequently, the cleaned data was exported to Stata version 16, for further management and statistical analysis. String variables were destringed and appropriately coded for statistical analysis. Given the predominantly categorical nature of the collected variables, any further categorization was guided by relevant literature, to ensure consistency with established research practices.

### Data analysis

Descriptive statistics were computed and reported. Frequencies and proportions were reported in tables and figures. The prevalence of illicit alcohol consumers was calculated as the proportion of participants who have reported ever-consuming illicit alcohol, within the specified age group (10–17 years) out of the total number of people reported as alcohol users, as show in the prevalence formula.


$$Prevalence=\;\frac{Participants\;ever\;reported\;consuming\;illicit\;alcohol}{Total\;number\;of\;participants\;reported\;as\;alcohol\;consumers}$$

Considering illicit alcohol consumption as the outcome (1 = illicit, 0 = non-illicit), a multiple logistic regression analysis, specifying for robust standard errors was performed to further assess the factors associated with illicit alcohol consumption. The utilization of robust standard errors bolstered the confidence in the validity of the estimated coefficients and their corresponding standard errors. This approach is particularly advantageous in scenarios where heteroscedasticity, the presence of outliers, or clustered data structures might be a concern. Consequently, the robustness of the statistical inferences was strengthened, leading to more reliable findings. To adjust for possible intra-cluster correlation at community-level, we used the variance-covariance estimator option specifying community as the cluster variable. Independent variables included age, sex, family size, alcohol-drinking behaviors and so on. Variables in the best-fit model were arrived at using both theoretical and empirical significance through the investigator-led stepwise regression approach (with p = 0.2 cut off point). The full and the nested model were fitted and compared using the Bayesian Information Criterion (BIC). The parsimonious model had lower BIC values and was picked as the best fit final model. The goodness of fit of the best fit logistic regression model was assessed using the Pearson Chi-square goodness of fit test, which indicated no evidence of poor fit (p = 0.389). Further, model performance was assessed using the area under the ROC curve, suggesting good estimation (AUROC = 0.7). All statistical analysis were done at a significance level of p-value < 0.05, using the 95% confidence level.

### Ethical consideration

This study was approved by UNZABREC (Ref: UNZA-4596/2023) and the National Health Research Authority. Authority to conduct the study within the district was obtained from the Ministry of Community Development. Informed consent was obtained from the parents or guardians (If available) of the underaged. However, considering that participants are underaged alcohol users with the majority living on the streets, it was challenging to locate their homes and parents. Therefore, in the absence of a parent or guardian for the participant, informed consent was obtained from a gatekeeper such as the area counselor of a specified community. Assent was obtained from all underage participants that were interviewed.

Interviews and discussions were conducted in a private place to ensure privacy and confidentiality. Participants were reminded that they were free to skip any questions if they preferred not to respond to any question without any consequences.

## Results

Three hundred and thirty-one (331) participants, consisting of 111 females and 220 males, aged between 10 and 17 years (with median age = 16 years, IQR: 15–17), were recruited for this study. Table 1 provides the background characteristics of the study participants. Most of the participants fall within the 16–17-years age groups 61.3% (203/331). The younger age groups (10–12 years) represented a smaller proportion 5.1% (17/331). In regards to sex, 66.5% (220/331) of participants were male, while 33.5% (111/331) were female (See Table 1).

**Table 1:** Demographic and Socio-economic characteristics of study participants (n = 331).

Demographic & socio-economic characteristics	*f* (%)
**Age (years)**	Median 16.0, IQR (15.1, 17.1)
10 - 12	17 (5.1)
13 - 15	111 (33.5)
16 - 17	203 (61.3)
**Sex**	
Female	111 (33.5)
Male	220 (66.5)
**Been to school**	
Yes	327 (98.8)
No	4 (1.2)
**Currently in School**	
Yes	221 (67.6)
No	106 (32.4)
**Level of Education**	
Grade 1–7	98 (30.0)
Grade 8–9	126 (38.5)
Grade 10–12	103 (31.5)
**Employment status**	
Employed	66 (19.9)
Unemployed	265 (80.1)
**Live with Biological parents**	
Yes	256 (77.3)
No	75 (22.7)
**Head of Household (HoH)**	
Father	137 (41.4)
Mother	96 (29.0)
Grandparent	44 (13.3)
Guardian	54 (16.3)
**HoH Education Level**	
Never been to school	14 (4.2)
Primary	73 (22.1)
Secondary	141 (42.6)
Tertiary	29 (8.8)
Don’t Know	74 (22.4)
**Head of Household Employment**	
Formal Employment	105 (31.7)
Informal Employment	156 (47.1)
Unemployment	70 (21.2)
**Family Size**	
Small (<5 members)	25 (7.6)
Medium (5–10 members)	62 (18.7)
Large (>10 members)	244 (73.7)
**Ever been imprisoned**	
No	277 (84.2)
Yes	52 (15.8)
**Engage in physical fights**	
No	142 (43.2)
Yes	187 (56.8)
**Use drugs**	
No	201 (60.7)
Yes	130 (39.3)
**Sexually Active**	
No	185 (55.9)
Yes	146 (44.1)
**Alcohol Drinking frequency**	
Daily	50 (15.1)
Weekly	183 (55.3)
Monthly	68 (20.5)
Yearly	30 (9.1)

CI-Confidence interval, cOR-Crude Odds Ratio, aOR-Adjusted Odds Ratio, (-) variables that did not make it in the best fit model.

Most of the participants had been to school, 98.8% (327/331), of which most had been up to grade 8–9 and above (38.5% and 31.5%) as their highest level of education attained. Further, 67.6% (221/331) reported being currently in school at the time of the study. The majority 80.1% (265/331) of these adolescents were unemployed. As shown on Table 1, 77.3% (256/331) of these adolescents indicated that they lived with their biological parents.

The majority of households were father-headed 41.4% (137/331), followed by mother-headed households 29% (96/331). Regarding education, 42.6% (141/331) of household heads had gone up to secondary school level (between grades 8–12) with 47% (156/331) working in the informal sector. The majority of participants, 73.7% (244/331) came from households with more than 10 members. Most adolescents, 84.2% (277/331) had never been imprisoned, yet 56.8%(187/331) reported involvement in physical fights. The majority did not use drugs 60.7% (201/331), and were not sexually active (55.9%, 185/331). More than half of these adolescents (55.3% (183/331) reported consuming alcohol on a weekly basis.

### Prevalence of illicit alcohol consumption among underage

**Fig 1:**
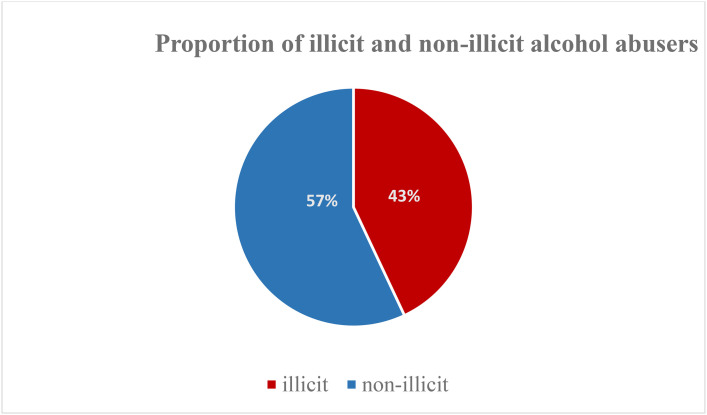
Proportion of illicit alcohol users among underaged adolescents in selected areas of Lusaka.

In the context of this study, illicit alcohol refers to alcoholic beverages produced, distributed, or sold outside the confines of legal regulations, or quality standards established by the Government of the Republic of Zambia, and/or governmental authorities. These beverages are frequently manufactured covertly, and may harbor contaminants or hazardous substances. A shown in Fig 1, among the underage adolescents who consume alcohol, an estimated 43% take illicit alcohol.

### Factors associated with illicit alcohol intake among adolescents

The univariate analysis showed that male adolescents (cOR 2.08, 95% CI: 1.20, 3.59, p = 0.008), sexually active adolescents alcohol users (cOR 1.99, 95% CI: 1.12, 3.55, p = 0.020), those that engage in physical fights (cOR 1.94, 95% CI: 1.02, 3.68, p = 0.044), recreational drug users (cOR 1.92, 95% CI: 1.13, 3.28, p = 0.017), and those ever been imprisoned (cOR 1.81, 95% CI: 1.09, 3.00, p = 0.022) had statistically significant higher odds of illicit alcohol consumption compared to their counterparts. Conversely, underage alcohol users with no form of employment had lower odds of illicit alcohol consumption (cOR 0.43, 95% CI: 0.23, 0.80, p = 0.007), compared to those that had some form of employment. Adolescents that drunk alcohol weekly (cOR 0.30, 95% CI: 0.12, 0.80, p = 0.015), monthly (cOR 0.56, 95% CI: 0.28, 1.12, p = 0.095), and yearly (cOR 0.27, 95% CI: 0.11, 0.66, p = 0.004) had lower odds of illicit alcohol consumption compared to those that drunk daily, with monthly consumption category not reaching statistical significance.

The adjusted analysis accounted for sex, employment status, drinking frequency, sexual activity, physical fights, use of drugs, ever imprisoned, and living condition. Adjusting for all these variables, males had 2.06 times higher odds of illicit alcohol intake compared to females (95% CI 1.23, 3.42 p = 0.005). Being unemployed was protective from illicit alcohol consumption (aOR 0.52, 95% CI 0.28, 0.99 p = 0.048). Further, as shown on Table 2, other variables including, drinking frequency, use of recreational drugs, being ever imprisoned were not statically significant predictors of f illicit alcohol consumption.

**Table 2:** Factors associated with illicit alcohol intake among adolescents in selected parts of Lusaka.

Variable	cOR (95% CI)	P-value	aOR (95% CI)	P-value
**Sex**				
Female	*Reference*			
Male	2.08 (1.20, 3.59)	0.008	2.06 (1.23, 3.42)	0.005
**Employment status**				
Employed	*Reference*			
Unemployed	0.43 (0.23, 0.80)	0.007	0.52 (0.28, 0.99)	0.048
**Drinking frequency**				
daily	*Ref*			
weekly	0.30 (0.12, 0.80)	0.015	0.40 (0.15, 1.11)	0.079
monthly	0.56 (0.28, 1.12)	0.095	0.68 (0.37, 1.25)	0.213
yearly	0.27 (0.11, 0.66)	0.004	0.43 (0.15, 1.22)	0.112
**Sexually active**				
No	*Reference*			
Yes	1.99 (1.12, 3.55)	0.02	1.36 (0.79, 2.36)	0.271
**Engaging in physical fights**				
No	*Reference*			
Yes	1.94 (1.02, 3.68)	0.044	1.41 (0.84, 2.39)	0.193
**Use drugs**				
No	*Reference*			
Yes	1.92 (1.13, 3.28)	0.017	1.20 (0.72. 1.99)	0.476
**Ever been imprisoned**				
No	*Reference*			
Yes	1.81 (1.09, 3.00)	0.022	1.12 (0.55, 2.28)	0.748
**Live with biological parents**				
No	*Reference*			
Yes	0.62 (0.31, 1.19)	0.151	0.63 (0.30, 1.34)	0.232

CI-Confidence interval, cOR-Crude Odds Ratio, aOR-Adjusted Odds Ratio, (-) variables that did not make it in the best-fit model.

## Discussion

This study examined the prevalence of illicit alcohol consumption and its associated factors among underage adolescents in peri-urban communities of Lusaka, Zambia. The findings indicate that almost half of adolescents who reported alcohol consumption were taking illicit alcohol, highlighting a substantial public health concern. This prevalence is higher than in previous studies conducted in the region [26,18], suggestive of the need for targeted interventions to protect adolescents in Lusaka. Being male and employment status were significant predictors of illicit alcohol consumption in both the crude and adjusted analyses. Additionally, older age, employment status, frequency of alcohol intake, sexual activity, involvement in fights, use of recreational drugs, and history of imprisonment were significant determinants of illicit alcohol consumption in the crude analysis. These findings align with previous research from Zambia and other countries [15,5,12,13].

As shown in the descriptive statistics, the age distribution of participants consuming illicit alcohol varied, reflecting the typical experimentation phase associated with adolescence. This aligns with previous studies documenting similar tendencies [27,28,29]. This developmental period often involves engagement in risky behaviors, including alcohol and drug use, as well as early sexual activity [30,28]. Our findings of early initiation into alcohol use among adolescents suggest a greater risk of alcohol-related problems, including abuse and dependence. The onset of alcohol use during this stage may escalate into heavier and more frequent consumption patterns. Therefore, the trajectory of alcohol consumption during adolescence is an important predictor of alcohol use patterns in adulthood [31]. Furthermore, alcohol use at early ages (10–13 years) significantly heightens the risk of progression to alcohol use disorders. This makes early intervention strategies that seek to delay the onset of drinking a reasonable and effective approach to preventing future alcohol-related problems [10,32]

The study’s findings reveal an alarming proportion of illicit alcohol consumption among underage adolescents in Lusaka, Zambia, resonating with prior studies that have reported similar trends across the country. However, this study shows a higher overall prevalence compared to previous studies in Lusaka [26,18]*.* Nearly half of adolescent alcohol users in this study reported consuming illicit alcohol. Despite ongoing efforts to curb this issue [33,7,18] including interventions such as Screening, Brief Intervention, and Referral to Treatment (SBIRT) [34,35], the persistence of this problem highlights the urgent need for tailored interventions targeting this vulnerable population.

Notably, this study reveals a sex disparity in illicit alcohol consumption, with a higher prevalence among males compared to females, consistent with broader research findings [36,37,38]. Although alcohol use has been reported among both genders, it appears to be more pronounced among male adolescents [39]. One possible explanation is that boys may drink more frequently than girls in order to conform to ideals of masculinity [40]. Another explanation is that adolescent males may be more susceptible to deviant peer pressure, seeking to align themselves with traditional gender role stereotypes that associate masculinity with toughness and autonomy. Additionally, biological factors such as higher alcohol tolerance among males may contribute to this disparity, whereas females tend to experience intoxication more quickly with the same quantity of alcohol. Nevertheless, it is important to note that despite lower consumption levels, females may develop alcohol dependence more rapidly than males. This has been described as the “telescoping” effect, whereby females progress more quickly from initial alcohol use to dependence and related problems, even while drinking less overall [41,42].

Notably, some studies have shown that alcohol use disorders escalate more rapidly into young adulthood in males than in females [43]. This suggests the need for interventions that challenge gender role stereotypes as a way to strengthen adolescent autonomy and promote healthy decision-making regarding alcohol consumption. The discrepancy underscores the importance of targeted interventions addressing male-specific risk factors contributing to alcohol abuse. Moreover, parents and schools should maintain consistent rules and consequences regarding adolescent drinking across genders, to avoid sending mixed messages about acceptable alcohol use for boys and girls [39].

Findings on employment status revealed that most illicit alcohol users in this study had some form of employment, suggesting a relationship between access to income and illicit alcohol consumption. The results indicate that unemployed adolescents were significantly less likely to engage in illicit alcohol intake compared to their employed counterparts. This finding is not surprising, as employment provides adolescents with financial resources that can be used to purchase alcohol, including illicit varieties. However, it contradicts other studies that have suggested a positive association between alcohol intake, binge drinking, and unemployment [Mangot-Sala, Smidt and Liefbroer, 2022,Saul, Lange and Probst, 2022,Haddad *et al.*, 2023]. This apparent contradiction can be better understood within the broader socio-economic context, particularly when considering the issue of child labor. Since this study focused on minors who are not legally permitted to buy, drink, or be employed, the results suggest that adolescents with access to income may resort to purchasing illicit alcohol, which is cheaper and more readily available in unregulated markets. Many adolescents earn money through temporary jobs, piecework, or scrap metal sales, providing them with a means to replenish funds easily. While such financial independence may help support household needs, it also opens up opportunities for risky behaviors, including alcohol and substance use [Van Hoof, 2019,Pantani, M. Sanchez and Pinsky, 2020]. Furthermore, employed adolescents may experience less parental or guardian supervision compared to their unemployed peers, increasing their opportunities to engage in risky behaviors, including illicit alcohol use [8]. This highlights the importance of parental involvement in monitoring adolescent behavior and addressing child labor within households, as this may reduce opportunities for illicit alcohol consumption [44,45,46,47,18,48,49,50,51,52].

This study has several limitations that warrant consideration. First, the cross-sectional design limits the ability to infer causal relationships between the identified factors and illicit alcohol consumption among adolescents. Without longitudinal data, it is difficult to determine whether these factors precede or result from illicit alcohol use. Second, reliance on self-reported data regarding alcohol consumption and related behaviors introduces the possibility of recall bias and social desirability bias, which may have led to under reporting or over reporting of alcohol use. Third, the exclusion of certain variables from the best-fit model due to multicollinearity or lack of statistical significance may have omitted potentially important influencing factors.

Despite these limitations, efforts were made to minimize potential biases through rigorous data collection procedures, including assurances of confidentiality to encourage honest reporting. Moreover, adjusting for confounding variables in the multivariable analysis strengthened the reliability of the findings by isolating the effects of individual factors on illicit alcohol intake.

## Conclusion

This study highlights a high prevalence of illicit alcohol consumption among underage adolescents in Lusaka, Zambia. Male gender and employment status emerged as the strongest predictors, while factors such as sexual activity, violence, drug use, and imprisonment were also linked to illicit alcohol use in crude analyses. Although not statistically significant, living with biological parents appeared to have a protective effect.

The findings suggests the need for sex- and age-specific interventions, particularly targeting adolescent males and those with income sources. Strengthening family bonds, creating supportive school and community environments, and providing targeted health education such as motivational interviewing are critical strategies for reducing illicit alcohol use among minors..
