# Correction: Prevalence of illicit alcohol consumption and associated factors among adolescents in selected communities of Lusaka, Zambia

**DOI:** 10.1371/journal.pone.0343512

**Published:** 2026-02-20

**Authors:** Dhally M. Menda, Tom Achoki, Bristol M. Ntebeka, Rosemary K. Zimba, Catherine M. Mulikita, Angela Rizzo, Maynards C. Tembe, Sharon Nkwemu, Rodgers Chilyabanyama, Karen Sichinga, Michael Kachumi, Choolwe Jacobs

Joel Msafiri Francis should not have been included in the author byline.
